# Clinical Significance of Germline Pathogenic Variants among 51 Cancer Predisposition Genes in an Unselected Cohort of Italian Pancreatic Cancer Patients

**DOI:** 10.3390/cancers14184447

**Published:** 2022-09-13

**Authors:** Alberto Puccini, Marta Ponzano, Bruna Dalmasso, Irene Vanni, Annalice Gandini, Silvia Puglisi, Roberto Borea, Malvina Cremante, William Bruno, Virginia Andreotti, Eleonora Allavena, Valentino Martelli, Fabio Catalano, Massimiliano Grassi, Maria Laura Iaia, Chiara Pirrone, Alessandro Pastorino, Giuseppe Fornarini, Stefania Sciallero, Paola Ghiorzo, Lorenza Pastorino

**Affiliations:** 1 Medical Oncology Unit 1, IRCCS Ospedale Policlinico San Martino, 16132 Genoa, Italy; 2 Department of Internal Medicine and Medical Specialties (DiMI), University of Genoa, V.le Benedetto XV 6, 16132 Genoa, Italy; 3 Department of Health Sciences (DISSAL), University of Genova, 16132 Genova, Italy; 4 Genetics of Rare Cancers, IRCCS Ospedale Policlinico San Martino, L.go Rosanna Benzi X, 16132 Genoa, Italy

**Keywords:** pancreatic cancer, genetics, DNA Damage Repair—Homologous Recombination Deficiency (DDR-HRD), hereditary cancer syndromes, germline, multigene panel testing, *BRCA*, *CDKN2A*, overall survival

## Abstract

**Simple Summary:**

Multigene germline panel testing data, extended beyond *BRCA*, in unselected pancreatic cancer patients, are missing in the Italian population. We aimed here to determine the prevalence and impact of pathogenic variants in 51 pancreatic cancer candidate susceptibility genes in an unselected cohort of Italian pancreatic cancer patients. We found that 17% were carriers. *CDKN2A* was the most frequently mutated gene, followed by *BRCA2* and *ATM*. Carriers showed better overall survival. A total of 41% of them had no family history. All *CDKN2A* carriers were older than 50 years, and *BRCA1/2* carriers were younger than 70 years. *CDKN2A* and *ATM* should be added to current *BRCA1/2* testing independently of family history in our population.

**Abstract:**

Multigene germline panel testing is recommended for Pancreatic Cancer (PC) patients; however, for non-*BRCA1/2* genes, the clinical utility is unclear. A comprehensive multi-gene assessment in unselected Italian PC patients is missing. We evaluated the prevalence and impact of Pathogenic Variants (PV) in 51 PC susceptibility genes in a real-world series of 422 Italian PC patients unselected for Family History (FH), compared the clinical characteristics and conducted survival analyses. 17% of patients had PVs (70/422), mainly in *BRCA1/2* (4.5%, all <70 y), *CDKN2A* (4.5%, all >50 y), ATM (2.1%). PV carriers were younger (64 vs. 67; *p* = 0.02) and had more frequent personal/FH of PC, melanoma and breast/ovarian cancer (all *p* < 0.05). The Overall Survival (OS) was longer in patients carrying PVs (HR 0.78; *p* = 0.090), comprising *ATM* carriers (HR 0.33; *p* = 0.054). In the oxaliplatin-treated subset, PV carriers showed better control of the disease, although this was not statistically significant (67% vs. 56%). CDKN2A, BRCA2 and ATM were the most frequently altered genes. *ATM* PVs were positively associated with OS in 41% of PV carriers, 60% of whom carried *CDKN2A,* *BRCA2* or *ATM* PVs, had negative FH and would have been missed by traditional referral. Thus, *CDKN2A* and *ATM* should be added to *BRCA1/2* testing regardless of FH.

## 1. Introduction

Exocrine Pancreatic Cancer (PC) is one of the deadliest solid tumors, with an average 5-year survival rate of 5–10% [1]. In 2020, 495,773 new cases and 466,003 deaths were observed globally (4.5% of all deaths caused by cancer) [2,3,4], and the incidence is expected to increase [5]. Pancreatic cancer remains one of the few cancers in which both early diagnosis and effective therapeutic processes have been lacking in recent years. Surgical resection is the only potentially curative treatment; however, less than 20% of patients present with resectable disease at the time of diagnosis [6,7].

Up to 10% of pancreatic cancer patients have familial inheritance [8]: about 3% develop pancreatic cancer in the context of hereditary cancer syndromes, and another 7% are classified as Familial Pancreatic Cancer (FPC), a definition attributed to individuals who have two or more first-degree relatives with pancreatic cancer. 

Hereditary pancreatic cancer syndromes include Hereditary Breast and Ovarian Cancer syndrome (HBOC), Peutz–Jeghers syndrome (SPJ), Familial Atypical Multiple Mole Melanoma (FAMMM)/melanoma-pancreatic cancer syndrome, Lynch syndrome (or Hereditary Non Polyposis Colorectal Carcinoma [HNPCC]), Familial Adenomatous Polyposis (FAP), ataxia-telangiectasia (ATM) and Hereditary Pancreatitis (HP). These hereditary cancer syndromes account for 10–15% of hereditary pancreatic cancers, while a genetic explanation for most FPCs has yet to be identified [9,10,11].

However, there are no established screening procedures for high-risk unaffected cases or for the general population, and it is unclear whether surveillance programs would provide clinical benefits [12]. Despite this lack of an internationally validated surveillance program for high-risk individuals, international guidelines (e.g., the National Comprehensive Cancer Network (NCCN) guidelines) recommend multigene germline testing for any patient with a confirmed PC diagnosis [13].

The implementation of this recommendation is hampered by the uncertainty around the clinical utility of multigene testing in pancreatic cancer patients, particularly for genes other than *BRCA1/2*. Indeed, Multi Gene Panel (MGP) testing results in a high rate of Pathogenic Variants (PVs), whose spectrum of cancer risks and penetrance is often not clearly defined. In addition, MGP testing generates high rates of Variants of Unknown Significance (VUS). These issues concern the medical community, as ambiguous findings may lead to unnecessary patient anxiety and unwarranted interventions [14].

In addition, the widespread availability of Next-Generation Sequencing (NGS) technologies, which has resulted in increased detection rate of PVs and VUS through MGP testing, has led to a paradigm shift also in cancer susceptibility testing [15]. Indeed, rather than submitting patients to pre-and post-genetic testing sessions, we were called to perform mainstream, oncologist-led, family history-independent genetic testing and refer for genetic counseling only those carrying a germline variant or with a strong clinical suspect of hereditary cancer syndrome [16]. 

For these reasons, while previously no more than 10% of PC cases were considered due to heritable factors, only a subset of which was explained by germline PVs in high-risk genes, recent sequencing efforts have revealed that as many as 15–20% of PC patients unselected for Family History (FH) bear PVs [17,18]. The use of MGP testing has also been implemented to detect druggable PVs (NCCN Guidelines 2022). The results from the POLO trial [19,20] confirmed the proof of principle of anti-PARP (Poly ADP-ribose polymerase) efficacy in PC patients harboring germline PVs in the *BRCA1/2* genes. 

In addition to ovarian [21], breast [22] and prostate cancer patients [23], the efficacy shown in this study extends the population to be tested and counseled for *BRCA* PVs to include PC patients. In addition, some other above mentioned hereditary syndromes associated with PC (i.e., Lynch Syndrome) are known to be caused by actionable mutations [24]. However, oncologists tend to focus on cancer treatment and overlook the implications of genetic counseling and diagnosis of hereditary syndromes. 

Thus, the POLO trial’s results have two main implications: (a) the need to select BRCA germline positive patients for personalized therapy and (b) the need to adequately counsel them for hereditary cancer syndromes. Of note, the US Food and Drug Administration (FDA), as well as the European Medicines Agency (EMA), approved Olaparib in this setting of patients based on POLO trial in 2019 [25] and 2020 [26] respectively, while the Italian Medicines Agency (AIFA) recently withdrew Olaparib from the approved treatments for PCs. 

The POLO study also confirmed the well-known platinum sensitivity of patients carrying germline PVs causing DNA Damage Repair—Homologous Recombination Deficiency (DDR-HRD), such as *BRCA1/2* [27]. However, a comprehensive analysis by MGP in unselected Italian PC patients is lacking. Preliminary data from different cohorts of unselected Italian PC patients focusing on *CDKN2A* and *BRCA1/2* showed a high prevalence of *CDKN2A* PVs, regardless of familial status [28] and of *BRCA1/2* PVs—these latter being observed only in patients < 74 y [29]. 

This study aimed to evaluate the prevalence and impact on outcomes of PVs in 51 PC susceptibility genes in a retrospective/prospective series of 422 Italian PC patients recruited at our Institution and unselected for FH.

## 2. Materials and Methods

### 2.1. Patients Selection and Characteristic

From 2020 to 2022, we recruited a consecutive series of 111 PC patients referred to our center for molecular analysis. Moreover, we retrospectively included an additional series of 311 non-consecutive PC patients who underwent molecular analysis at our center. Patients with neuroendocrine pancreatic tumors were excluded. All patients (N = 422) were unselected for age, FH or personal history, and there were no differences in terms of patients’ characteristics (age, family, personal history and prevalence of PVs) between retrospective and prospective groups, except for the stage and Overall Survival (OS).

This may be explained by stage migration and better management of patients in recent years. All patients signed an informed consent according to local ethics committee protocols and answered a standardized family history questionnaire on the presence and type of cancers in the family. One hundred twenty-two patients have been described in previous publications; however, the analysis was limited to *CDKN2A* or *BRCA1-2* [28,30], as well as Mismatch Repair (MMR) genes for a subset of high-risk patients [30,31]. In addition, 31 patients, either positive for *CDKN2A* PVs or negative but considered high-risk because of PC family history, had been previously tested by Whole-Exome Sequencing (WES) [32] and were re-tested here by MGP.

### 2.2. Gene Panel

Next-Generation Sequencing (NGS) was performed for all 422 patients using an Ion Custom Panel (125–275 bp amplicon target sizes; 355 kb; 1266 amplicons), including 51 genes involved in DDR, PC susceptibility and hereditary pancreatitis (*APC*, *ARID1A, ARID1B*, *ARID2*, *ATM*, *ATR*, *ATRX*, *BAP1*, *BARD1*, *BMPR1A*, *BRCA1*, *BRCA2*, *BRIP1*, *CDC73*, *CDK12*, *CDK4*, *CDKN2A*, *CHEK1*, *CHEK2*, *COL7A1*, *CPA1*, *EPCAM*, *ERCC4*, *FAM175A*, *FANCA*, *FANCB*, *FANCC*, *FANCD2*, *FANCE*, *FANCF*, *FANCG*, *FANCL*, *MLH1*, *MRE11*, *MSH2*, *MSH6*, *NBN*, *PALB2*, *PMS2*, *PRSS1*, *RABL3*, *RAD50*, *RAD51*, *RAD51B*, *RAD51C*, *RAD51D*, *RET*, *SPINK1*, *STK11*, *SUFU* and *TP53*) according to the recent literature [33,34]. 

The NGS libraries were constructed using the Ion AmpliSeq Kit for Chef DL8 (Thermo Fisher Scientific, Waltham, MA, USA), according to the manufacturer’s protocol starting from 20 ng of genomic DNA. According to the manufacturer, the libraries were sequenced with an Ion Torrent S5 GeneStudio Plus system using 530 Chip (Thermo Fisher Scientific). The S5 sequencing data were analyzed by the Ion Torrent Software Suite (Thermo Fisher Scientific) using the plugin Variant Caller and Ion Reporter (Thermo Fisher Scientific). The filtered variants were analyzed and classified according to the American College of Medical Genetics and Genomics (ACMG) classification [35]. Only ACMG class 4 (Likely Pathogenic) and 5 (Pathogenic) variants were considered as PVs in this study.

### 2.3. Statistical Analysis 

Descriptive characteristics were reported as mean (standard deviation) or absolute frequency (relative frequency). Patients carrying PVs were compared to the non-carriers using T-test for continuous variables and Chi-squared or Fisher’s exact test for categorical variables. Univariable Cox proportional hazards models were used to identify characteristics associated with prognosis in terms of overall survival. Subsequently, we presented multivariable Cox proportional hazards models adjusting for confounders. Additionally, as a sensitivity analysis, age-adjusted Cox proportional hazards models were performed to specifically assess the impact of each type of mutation. 

Within the subsample of patients who received oxaliplatin-based treatment, the association between PVs and disease progression was evaluated by a Chi-squared test. Finally, an age-adjusted Cox proportional hazards model was performed to compare responding and not-responding patients. All statistical analyses were conducted using Stata version 16.0 (Stata Corporation, College Station, TX, USA), and *p*-values < 0.05 were considered statistically significant.

## 3. Results

We recruited 422 patients belonging to a real-world series of patients diagnosed with Pancreatic cancer, unselected for family history and analyzed them using a multigene panel containing 51 genes. Table 1 shows the patients’ characteristics according to the germline status. 

Overall, 52% were females, and the mean age was 67 years (range 30–92). As expected, germline PV carriers were significantly younger than non-carriers (*p*-value = 0.0254). Overall, 13% of patients had a first or second-degree relative with PC. A family history of PC was more frequent in carriers than in non-carriers (26% vs. 11%; *p* = 0.001); the same pattern was confirmed in cases with a personal and/or family history of either breast and ovarian cancer (36% vs. 14%, *p* < 0.001), melanoma (19% vs. 5%; *p* < 0.01) and personal history of other tumors (32% vs. 13%, *p* < 0.001).

### 3.1. Prevalence of Pathogenic Variants in Pancreatic Cancer Susceptibility Genes: Correlation with Family History and Age at Diagnosis

**Figure 1:**
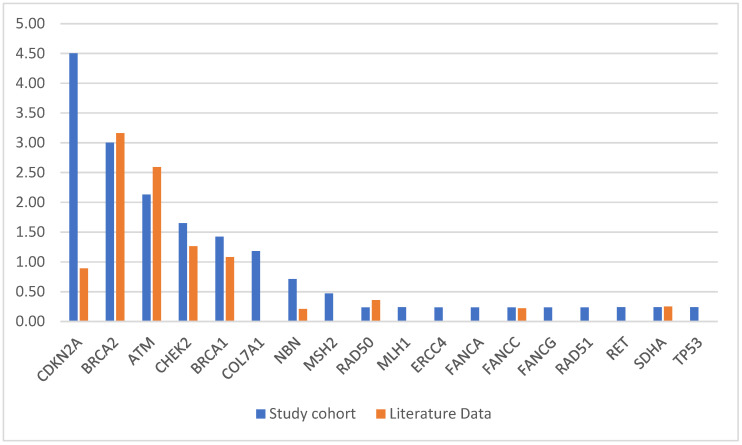
Comparison between the relative frequency of candidate susceptibility genes PVs in our cohort and literature data according to the systematic review by Astiazaran-Symonds E and Goldstein AM [37].

Seventy out of 422 enrolled patients (17%) carried one or more pathogenic variant (PV) in at least one of the 51 genes included in the panel (Table 1). *CDKN2A* was the most frequently mutated gene (19/422; 4.5%), confirming the founder p.Gly101Trp as the most frequent PV in our series (11/19 = 57.9%) [36]. For this reason, *CDKN2A* in our cohort shows a higher frequency compared to the literature data [37] (Figure 1). 

*BRCA2* (13/422 = 3.1%) was the second most frequently mutated gene. *BRCA1/2* positive patients (19/422; 4.5%) were as frequent as those carrying *CDKN2A* PV/LPVs. The third most frequently mutated gene was *ATM* since PV/LPVs were detected in nine patients (2.1%). Altogether, PV/LPVs in these four genes were 50% of all mutations in our cohort (37/74). Notably, 41% (29/70) of patients carrying PV/LPVs had no pancreatic, breast or melanoma personal and/or family history (Table 2).

The prevalence of PVs in HRD pathway-related genes (*ATM*, *BRCA1/2*, *CHEK2*, *ERCC4*, *FANCA*, *FANCC*, *FANCG*, *NBN*, *RAD50* and *RAD51*) was 10.4%. The inclusion of HR-DDR pathway-related genes compared to *BRCA1/2* alone more than doubled the prevalence of pathogenic variants (10.4% vs. 4.5%). Among these PVs, 41% (18/44) were found in sporadic patients.

Three patients showed multiple PVs: two sporadic PC patients had two (in *BRCA2* and *CHEK2*) and three (in *ATM*, *CDKN2A* and *NBN*) pathogenic variants, respectively. The third, who developed both melanoma and PC, showed 2 PVs (in *CDKN2A* and *NBN*). Interestingly, two out of three patients carrying *NBN* PVs had a concomitant PV in a high penetrance predisposition gene (*CDKN2A* or *ATM*).

Seven PVs were found in *CHEK2* (1.7%), four of whom had no FH. Five PVs (1.2%) were found in the *COL7A1* gene; among these patients, only two showed a family history of PC and breast cancer. Finally, we detected two PVs in *MSH2*, in 2 patients with suspected Lynch Syndrome. No variants were identified in the *PALB2* gene. The complete list of LPs and PVs is described in Supplementary Table S1.

As expected, PVs in *BRCA1/2* were more frequent in subjects with a personal and/or family history for breast/ovarian cancer (12/76, 15.8%). However, a 1.8% frequency was observed even in sporadic subjects (5/283). *CDKN2A* PVs were frequent in patients with a family history of PC (8/55, 14.5%) and with a personal or family history of melanoma (6/30, 20%); however, the rate remains high (8/283, 2.8%) in sporadic subjects. Notably, ATM PVs frequency was 5.4% in patients with a family history of PC (3/55) and 3.3% in patients with personal or family history of melanoma (1/30). *ATM* PVs mutation frequency was as high as that of *BRCA-2* (1.8%) in sporadic patients. 

Overall, 11.3% of PV were found in sporadic patients (32/283) and would have been missed if we tested patients based only on family history (Table 2). When we analyzed age at diagnosis comparing patients according to specific PVs, we found that the overall frequency of PVs was 22.2% among patients ≤50 y, 24.4% among patients 51–60 y, 17.5% among patients 61–70 y and 11.6% for those who were >70 y. The highest frequency of *BRCA1/2* PV was found among the youngest patients (≤50, 13.9%), while no PVs were found in patients older than 70.

Conversely, the highest *CDKN2A* mutation rate was found in patients aged 61–70 y (6.3%) and was 3.8% in patients > 70 y. No PVs in *CDKN2A* were found in patients younger than 50 y (Supplementary Table S2). Overall, patients with *BRCA1/2* genes were younger (mean age = 58) than *CDKN2A* patients (mean age = 67) (Supplementary Table S2).

### 3.2. Impact of Germline Pathogenic Variants on Patients’ Survival

Table 3 summarizes patients characteristics according to OS. Median follow-up was 9.7 months (interquartile range: 4.3–21,25) and median OS was 11.5 months (range 10.4–13.3). In univariable analysis, as expected, 10-years increase in age was associated with worse prognosis (HR 1.18; 95% CI 1.06–1.32; *p* = 0.004), as well as metastatic disease compared to resectable stage (HR 3.20; 95% CI 2.38–4.29; *p* < 0.001). These results were confirmed in multivariable analysis (age: HR 1.18; 95% CI 1.06–1.33; *p* = 0.004) (metastatic disease: HR 3.39; 95% CI 2.51–4.57; *p* < 0.001). 

Patients carrying any PVs showed better OS compared to non-carriers in the univariable analysis (HR 0.78; 95% CI 0.59–1.04; *p* = 0.090) and was confirmed in the multivariable analysis (HR 0.81; 95% CI 0.61–1.09; *p* = 0.160). In the sensitivity analysis we also evaluated the impact of PVs in single gene and we found that all nine patients carrying *ATM* PVs had a better prognosis compared to non-carriers in terms of median OS (15 vs. 11 months; age-adjusted HR 0.33; 95% CI 0.10–1.02; *p* = 0.054). No significant differences were observed between patients with PVs in other genes and non-carriers.

### 3.3. Impact of Germline Pathogenic Variants on Response to Oxaliplatin

A total of 106 patients received oxaliplatin-based treatment: 25 (34%) in the neoadjuvant and 81 (76%) in the first- or second-line metastatic setting. Overall, in the neoadjuvant setting, 24% (N = 6) had a Progression of Disease (PD) as the Best Response (BR) to oxaliplatin, 28% (N = 7) had a partial response and 32% (N = 8) had stable disease. In the metastatic setting, 42% (N = 34) had a PD as the best response to oxaliplatin, 21% (N = 17) had a partial response, and 30% (N = 24) had a stable disease. Patients carrying a PV more frequently showed a good response to treatment (67%) compared to non-carriers (56%) but differences were not statistically significant (*p* = 0.381). When we focused on patients with *BRCA* or *ATM* PVs, all nine patients showed control of disease as BR to oxaliplatin. 

## 4. Discussion

We analyzed a panel of 51 genes in a retrospective/prospective cohort of 422 Italian PC patients unselected for family history, stage or age of onset. To the best of our knowledge, this is the largest cohort of unselected PC Italian patients analyzed with a MGP comprehensive of all PC susceptibility genes described to date. Overall, 70 PVs (17%) were found, a relatively high frequency compared to similar studies. However, due to the heterogeneity in cohort selection and dissimilar sequencing approaches across studies, the prevalence of PVs in PC susceptibility genes cannot be easily quantified, ranging from 5% to nearly 20% in different studies and reviews [18,34,37,38,39,40,41,42]. 

Here, we confirm in an extended cohort of Italian PC patients, the high *CDKN2A* PV/LPV rate previously described [28,43], finding a total *CDKN2A* mutation rate of 4.5% (19/422), as high as the one conferred by *BRCA1* and *BRCA2* together (BRCA1 n = 6/422 and *BRCA2* 13/422). According to the literature, *CDKN2A* germline variants in patients unselected for FH range from 0.1 to 2.6% [37,44,45,46]. In our study, the rate increased to 14.5% (n = 8/55) and 20% (n = 6/30) in subjects with familial PC or with a personal and/or FH of melanoma, respectively. 

The latest frequency confirms recent findings from an extended melanoma family study in the Italian population showing a 12.4% of PVs in melanoma families with PC cases [47]. However, the mutation rate remains high (2.8%) even in sporadic subjects. This may reflect the presence of founder *CDKN2A* PVs in our population [36].

As expected, *BRCA1* and *BRCA2* PV rate nearly doubled in cases with a FH of PC nearly compared to sporadic subjects (3.6% vs. 1.7%), while it ranged from 1.7% to 15.8% in subjects with a personal or family history of breast/ovarian cancer (Table 2). Again, these data confirm previously observed *BRCA1/2* mutation rates in Italian families with history of both breast/ovarian and PC [40,41]. 

Carriers of *BRCA1/2* PVs showed a lower age of onset compared to *CDKN2A* carriers. While *CDKN2A* PVs are distributed only in the age categories above 50 years, *BRCA1/2* are the most common PVs in the age category below 50 years (13.9%). In our cohort, the oldest patient carrier of a germline *BRCA1*/*2* PVs was 70 years old. This supports the results obtained in a different unselected cohort of Italian PC patients tested for *BRCA1/2* only, showing that *BRCA1/2* mutations were found in patients < 74 y [29].

*ATM* was the first most frequently mutated gene in the age category between 51 and 60 years and the third mutated gene overall (2.13%). Interestingly, *ATM* PV/LPVs frequency was second to *CDKN2A* only in patients with a family history of PC (5.4%) and was found at a frequency of 3.3% in patients with familiarity for melanoma. These data also confirm recent figures observed for *ATM* germline PVs in melanoma cases [47,48,49]. 

Overall, *CHEK2* and *COL7A1* PVs were found with a frequency above 1%. Further investigation will be needed to elucidate their role in pancreatic cancer predisposition. However, our findings are in line with those from the PAN Cancer [34]. A recent paper found that the combined frequency of heterozygous carriers of *CHEK2* in the general population was 1.2% [50]. Interestingly, PVs in *TP53* and MMR genes were below 1% and were found in patients with a personal and/or FH of breast/ovarian and colon cancer, respectively.

In our study, we observed that patients carrying any PVs had a better OS compared to non-carriers (HR = 0.66; 95% CI 0.47–0.93; *p* = 0.017). However, when we focused on PVs in specific genes, only patients carrying *ATM* PVs (N = 9) showed better OS compared to WT patients (N = 345) (HR = 0.33). Our results confirm previous reports that suggested *ATM* alterations as prognostic for improved outcomes in patients with PC [51]. 

Despite efforts in extensive molecular profiling in PC for actionable genetic alterations or pathways, targeted therapy showed to provide limited clinical benefit for patients with this disease to date [52]. One exception is alterations in DNA damage repair (DDR) genes, especially *BRCA1/2*, since BRCA-associated tumors, including breast, prostate and ovarian tumors, respond to poly–(adenosine diphosphate–ribose) polymerase (PARP) inhibitors and platinum-based therapy. 

However, the efficacy of PARP inhibitors in DDR-related genes other than *BRCA* is less clear. Recent two phase II nonrandomized clinical trials showed that Olaparib was well tolerated. However, it showed limited antitumor activity in patients with advanced, platinum-sensitive PC with DDR-genetic alterations, most of them carried *ATM* alterations [53]. Unfortunately, no patients with *ATM* alterations were treated with PARP inhibitors in our cohort. However, we observed a trend in better disease control in PV carriers treated with oxaliplatin, although not statistically significant. 

More data in larger cohorts are needed to confirm these results. Although the prevalence of DNA MMR is <1%, these patients may benefit from immunotherapy [24], thus we believe that evaluation of MMR system (somatic and/or germline) is crucial in PC patients as well as in all other solid tumor types [54]. *BRCA1/2*, *CDKN2A* and *ATM*, account for an overall PV/LPV prevalence of 11.1% in our cohort.

Had we selected PC patients for genetic testing according to their personal and family history suggestive for familial PC or hereditary cancer syndromes, we would have missed 41% of patient carriers of PVs in candidate gens. Sixty% of them were carriers of PVs in *CDKN2A*, *BRCA2* and ATM. At least *CDKN2A* and *ATM*, should be added to *BRCA1/2* testing by MGP independently of FH. 

These PVs are clinically actionable since they result in hereditary cancer syndromes for which international guidelines support surveillance aimed to reduce mortality among relatives. Although such guidelines are not of proven efficacy for PC mortality reduction, identification of probands may still reduce mortality for other cancers through ‘ad hoc’ prevention protocols. However, several studies and meta-analyses have shown that the diagnostic yield of pre-malignant or malignant lesions (including earlier stage) in high risk individuals undergoing screening/surveillance is much higher than the lifetime risk of PC in the general population and this might be considered a surrogate for mortality reduction [55,56,57,58,59].

In Italy, the AISP (Italian Association for the Study of the Pancreas), developed a registry of families at high risk of PC to be included in surveillance protocols [59,60]. As previously mentioned, an extended MGP testing—beyond *BRCA1* and *2*—in all PC patients in not standard of care in Italy and an estimate of a real world MGP frequency in unselected PC patients was missing. The registry is currently recruiting families at high-risk because of family history or positive mutation testing. Our data support an extended MGP testing (at least to *CDKN2A* and *ATM*) to identify positive high-risk carriers to be included in registry surveillance protocols, also independently of family history. 

We acknowledge that our study presents some limitations, mainly because of a non- consecutive and retrospective enrollment of part of the cohort. In addition, few data are available on treatments (oxaliplatin-based, anti-PARP and/or immunotherapy) in PV carriers. No correlation with somatic tumor samples was conducted. Moreover, stage migration and changes in patient management were observed during the long patient recruitment period. 

## 5. Conclusions

In conclusion, our study showed that the universal use of MGP testing in PC patients allowed us to identify a high PV prevalence (17%). These data might address unmet clinical need to identify actionable mutations and provide the opportunity to identify well-known hereditary cancer syndromes. Finally, 40% of patients with PVs did not have suspected criteria within our cohort and would have been missed with standard selection criteria. 

Thus, we propose extending the offer of genetic testing beyond *BRCA1* and *2* at least to *CDKN2A* and *ATM*, independently of family history, with no age restrictions to identify mutations and actionable not only for target therapy in affected members but also for surveillance research protocols in carriers, including unaffected relatives. 

## Figures and Tables

**Table 1:** Characteristics of the study cohort according to the germline status.

	Overall (N = 422)	Non-Carriers (N = 352)	PV Carriers (N = 70)	*p*-Value
Age, mean (range)	67 (30–92)	67.4 (30–92)	64.3 (44–82)	**0.0254**
Female, N (%)	221 (52)	189 (54)	32 (46)	0.222
Initial stage, N (%)				
Resectable	92 (22)	76 (22)	16 (23)	0.323
Borderline	25 (6)	21 (6)	4 (6)	
Unresectable	51 (12)	38 (11)	13 (19)	
Metastatic	188 (45)	158 (45)	30 (43)	
Missing	66 (16)	59 (17)	7 (10)	
FH for PC, N (%)	55 (13)	37 (11)	18 (26)	**0.001**
Personal/FH for BC/Ovarian, N (%)	76 (18)	51 (14)	25 (36)	**<0.001**
Personal/FH for melanoma, N (%)	30 (7)	17 (5)	13 (19)	**<0.001**
Other primary cancers, N (%)	64 (16)	43 (13)	21 (32)	**<0.001**

Pts = patients; wt = wild-type; PV = pathogenic variant/likely pathogenic variant; FH = family history. PC = pancreatic cancer; BC = breast cancer; significant values are shown in bold.

**Table 2:** Pathogenic variant frequency: overall and according to personal and family history.

Genes	Cumulative Frequency		FH of PC		Personal and/or FH of BC and Ovary		Personal and/or FH of Melanoma		Frequency in Sporadic PC		
	n = 422		n = 55		n = 76		n = 30		n = 283		
	n	%	n	%	n	%	n	%	n	%	
*ATM*	9	(2.1)	3	(5.4)	1	(1.3)	1	(3.3)	5	(1.7)	*
*BRCA1*	6	(1.4)	1	(1.8)	5	(6.6)	1	(3.3)	-	-	**
*BRCA2*	13	(3)	1	(1.8)	7	(9.2)	2	(6.7)	5	(1.7)	*/**
*BRCA1/2*	19	(4.5)	2	(3.6)	12	(15.8)	3	(10)	5	(1.7)	*/**
*CDKN2A*	19	(4.5)	8	(14.5)	5	(6.6)	6	(20)	8	(2.8)	*/**
*CHEK2*	7	(1.6)	1	(1.8)	-	-	1	(3.3)	5	(1.7)	
*COL7A1*	5	(1.2)	1	(1.8)	1	(1.3)	-	-	3	(1)	
*ERCC4*	1	(0.2)	-	-	-	-	-	-	1	(0.3)	
*FANCA/C/G*	3	(0.7)	2	(3.6)	2	(2.6)	1	(3.3)	-	-	**
*MLH1*	1	(0.2)	-	-	1	(1,3)	-	-	-	-	
*MSH2*	2	(0.4)	-	-	1	(1.3)	-	-	1	(0.3)	
*NBN*	3	(0.7)	-	-	1	(1.3)	1	(3.3)	2	(0.7)	*
*RAD50/51*	2	(0.4)	1	(1.8)	1	(1.3)	1	(3.3)	-	-	**
*RET1*	1	(0.2)	-	-	-	-	-	-	1	(0.3)	
*SDHA*	1	(0.2)	-	-	-	-	-	-	1	(0.3)	
*TP53*	1	(0.2)	-	-	1	(1.3)	-	-			
All genes	74	(17.5)	18	(32.7)	26	(34.2)	14	(46.7)	32	(11.3)	

FH = family history; PC = pancreatic cancer; BC = breast cancer. * 2 or more variants in the same patient; ** some patients were included in more than one category.

**Table 3:** Cox proportional hazards models to identify characteristics associated with prognosis in terms of overall survival.

	Univariable Cox Models		Multivariable Cox Model N = 340	
	HR (95% CI)	*p*-Value	HR (95% CI)	*p*-Value
Age (10-years) ***	1.18 (1.03–1.32)	**0.004**	1.18 (1.06; 1.33)	**0.004**
Male vs. Female *	1.06 (0.85–1.31)	0.612	1.13 (0.91; 1.40)	0.270
Initial stage (N = 353) **				
Resectable	1.00	Ref	1.00	Ref
Borderline	1.24 (0.66–2.30)	0.504	1.25 (0.66; 2.39)	0.491
Unresectable	1.39 (0.93–2.08)	0.113	1.39 (0.92; 2.09)	0.116
Metastatic	3.20 (2.38–4.29)	**<0.001**	3.39 (2.51; 4.57)	**<0.001**
FH for PC *	0.85 (0.62–1.17)	0.315	0.90 (0.65; 1.24)	0.526
Personal/FH for BC/Ovarian *	0.91 (0.69–1.21)	0.519	0.92 (0.69; 1.22)	0.552
Personal/FH for melanoma *	0.99 (0.66–1.49)	0.969	1.08 (0.71; 1.63)	0.723
Other primary cancers, (N = 395) *	0.76 (0.56–1.04)	0.089	0.74 (0.54; 1.01)	0.061
Presence of any PV vs. WT *	0.78 (0.59–1.04)	0.090	0.81 (0.61; 1.09)	0.160

Multivariable estimates are adjusted for * age, ** age, sex and PV, *** sex. FH = family history; WT = wild-type; PV = Pathogenic Variant/Likely Pathogenic Variant; PC = pancreatic cancer; BC = breast cancer; significant values are shown in bold.

## Data Availability

The data presented in this study are available on request from the corresponding author.

## Supplementary Materials

The following supporting information can be downloaded at: https://www.mdpi.com/article/10.3390/cancers14184447/s1, Table S1: List of Likely Pathogenic and Pathogenic Variants; Table S2: Overall mutation rate and related to a single gene.
