# Supplementary material for: Clinical Significance of Germline Pathogenic Variants among 51 Cancer Predisposition Genes in an Unselected Cohort of Italian Pancreatic Cancer Patients
Source: Cancers (Basel). 2022 Sep 13;14(18):4447. doi: 10.3390/cancers14184447 (PMC9496779; doi:10.3390/cancers14184447)
Supplement: Supplementary file 1 [file cancers-14-04447-s001.zip › cancers-1881458-supplementary.pdf]

cancers MDPI

Supplementary

Clinical Significance of Germline Pathogenic Variants among 51 Cancer Predisposition Genes in an Unselected Cohort of Italian Pancreatic Cancer Patients

Table S1. List of Likely Pathogenic and Pathogenic Variants.

Genes	REF SEQ	Coding	Amino Acid Change	Variant Effect	ACMG	N°
ATM	NM_000051 .3	c.103C>T	p.Arg35Ter	nonsense	P	1
		c.331+2T>G	p.?	splicing	P	1
		c.1212_1213delGA	p.Asn405Ter	nonsense	P	1
		c.2125-1G>T*	p.?	splicing	LP	1
		c.2128delA*	p.Thr710Glnf-sTer25	frameshiftDeletion	LP	1
		c.2502_2503insA	p.Val835Serf-sTer7	frameshiftInsertion	P	1
		c.3802delG	p.Val1268Ter	nonsense	P	1
		c.3894_3895insT	p.Ala1299Cys-fsTer3	frameshiftInsertion	P	1
		c.4451delT	p.Met1484Argf-sTer15	frameshiftDeletion	P	1
		c.6658C>T	p.Gln2220Ter	nonsense	P	1
BRCA1	NM_007294 .3	c.(80+1_81-1)_(134+1_135-1)del	-	deletion	P	1
		c.514delC	p.Gln172Asnf-sTer62	frameshiftDeletion	P	1
		c.1380_1381insA	p.Phe461IlefsTer1 9	frameshiftInsertion	P	1
		c.1687C>T	p.Gln563Ter	nonsense	P	1
		c.3875_3878delGTCT	p.Ser1292LeufsTe r14	frameshiftDeletion	P	1
		c.5027_5030delTAAC	p.Thr1677IlefsTer 2	frameshiftDeletion	P	1
BRCA2	NM_000059 .3	c.204delA	p.Lys68Asnf-sTer12	frameshiftDeletion	P	1
		c.2542A>T	p.Lys848Ter	nonsense	LP	1
		c.3545_3546delTT	p.Phe1182Ter	nonsense	P	1
		c.3881T>G	p.Leu1294Ter	nonsense	P	1
		c.5723_5724delTA	p.Leu1908Argf-sTer2	frameshiftDeletion	P	1
		c.5796_5797del	p.His1932Glnf-sTer12	frameshiftDeletion	P	1
		c.6468_6469delTC	p.Gln2157IlefsTer 18	frameshiftDeletion	P	1
		c.6591_6592delTG	p.Glu2198Asnf-sTer4	frameshiftDeletion	P	1
		c.7007+5G>A	p.?	splicing	LP	1
		c.7180A>T	p.Arg2394Ter	nonsense	P	1
		c.8057T>C	p.Leu2686Pro	missense	P	1
		c.8594T>A	p.Leu2865Ter	nonsense	P	1

		c.9498delT	p.Glu3167Argf-sTer50	frameshiftDeletion	P	1
CDKN2A	NM_000077.5	c.-201_-198delinsctt	-	frameshiftDeletion	LP	2
		c.79G>T	p.Glu27Ter	nonsense	P	2
		c.194T>C	p.Leu65Pro	missense	LP	1
		c.202_203delGCinsTT	p.Ala68Leu	missense	LP	1
		c.249C>A	p.His83Gln	missense	LP	1
		c.301G>T	p.Gly101Trp	missense	P	11
		c.377T>A	p.Val126Asp	missense	LP	1
CHEK2	NM_007194.4	c.85C>T	p.Gln29Ter	nonsense	P	1
		c.349A>G	p.Arg117Gly	missense	P	1
		c.475T>C	p.Tyr159His	missense	LP	2
		c.483_485delAGA	p.Glu161del	disruptive_in-frame_deletion	LP	1
		c.1427C>T	p.Thr476Met	missense	LP	2
COL7A1	NM_000094.3	c.7344+2T>C	p.?	splicing	LP	1
		c.497_498insA	p.Val168Glyf-sTer12	frameshiftInsertion	P	1
		c.5047C>T	p.Arg1683Ter	nonsense	P	1
		c.1A>G	p.Met1?	missense	LP	1
		c.497_498insA	p.Val168Glyf-sTer12	frameshiftInsertion	P	1
ERCC4	NM_005236.2	c.886C>T	p.Gln296Ter	nonsense	LP	1
FANCA	NM_000135.3	c.3391A>G	p.Thr1131Ala	missense	P	1
FANCC	NM_000136.2	c.520C>T	p.Arg174Ter	nonsense	P	1
FANCG	NM_004629.1	c.601_602delCA	p.Gln201Glyf-sTer33	frameshiftDeletion	LP	1
MLH1	NM_000249.3	c.1410-2A>G	p.?	splicing	P	1
MSH2	NM_000251.2	c.528_529delTG	p.Cys176Ter	nonsense	P	1
	NM_000251.2	c.(1386+1_1387-1)_(1510+1_1511-1)del	-	deletion	P	1
NBN	NM_002485.4	c.1601_1602insA	p.Asn534Lys-fsTer5	frameshiftInsertion	LP	1
		c.2071-2A>C	p.?	splicing	P	1
		c.2140C>T	p.Arg714Ter	nonsense	P	1
RAD50	NM_005732.3	c.3715C>T	p.Arg1239Ter	nonsense	P	1
RAD51	NM_002875.5	c.220_221dupAT	p.Leu75Phef-sTer2	frameshiftInsertion	LP	1
SDHA	NM_004168.3	c.553C>T	p.Gln185Ter	nonsense	P	1
TP53	NM_000546.5	c.365_366delTG	p.Val122Aspf-sTer26	frameshiftDeletion	P	1
RET	NM_020975.5	c.1846_1848delGAG	p.Glu616del	nonframeshiftDeletion	LP	1

Table S2. Mutation rate according to age.

**Age**	**≤ 50**		**51-60**		**61-70**		**>70**	
	n=36 (8,53%)		n=78 (18,48%)		n=126 (29,86%)		n=181 (42,89%)	
ATM	1	2,78%	5	6,41%			3	1,66%
BRCA1	2	5,56%	1	1,28%	3	2,38%		
BRCA2	3	8,33%	4	5,13%	6	4,76%		
BRCA1/2	5	13,89%	5	6,41%	9	7,14%		
CDKN2A			4	5,13%	8	6,35%	7	3,87%
CHEK2			3	3,85%	2	1,59%	2	1,10%
COL7A1					2	1,59%	3	1,66%
ERCC4	1	2,78%						
FANCA/C/G			1	1,28%			2	1,10%
MLH1					1	0,79%		
MSH2	1	2,78%	1	1,28%				
NBN			2	2,56%			1	0,55%
RAD50/51							2	1,10%
RET1					1	0,79%		
SDHA							1	0,55%
TP53							1	0,55%
Overall Mutation Rate	8	22,22%	19	24,36%	22	17,46%	21	11,60%
